# Stress Studies of Tenofovir Disoproxil Fumarate by HPTLC in Bulk Drug and Pharmaceutical Formulation

**DOI:** 10.1100/2012/894136

**Published:** 2012-04-19

**Authors:** Shweta Havele, Sunil R. Dhaneshwar

**Affiliations:** Research and Development Centre in Pharmaceutical Sciences and Applied Chemistry, Poona College of Pharmacy, Bharati Vidyapeeth University, Erandwane, Pune 411038, India

## Abstract

A stability-indicating high-performance thin-layer chromatographic (HPTLC) method for determination of tenofovir disoproxil fumarate in bulk drug and in tablet has been developed and validated. The mobile phase selected was chloroform : methanol (9.0 : 1.0, v/v) with ultraviolet (UV) detection at 260 nm. The retention factor was found to be 0.49 ± 0.03 with correlation coefficients of 0.9994 in the range 300–1500 ng/spot and with an accuracy of 99.25%. Method had the potential to determine tenofovir disoproxil fumarate from tablet without any interference, and it was a stability-indicating one.

## 1. Introduction

The aim of the present study was to establish the inherent stability of tenofovir disoproxil fumarate through stress studies under a variety of International Conference on Harmonisation (ICH) recommended test [1–3] and to develop a stability-indicating assay [4–6].

Tenofovir is converted intracellularly to the diphosphate. This diphosphate halts the DNA synthesis of HIV through competitive inhibition of reverse transcriptase and incorporation into viral DNA. It also inhibits hepatitis B virus polymerase, resulting in inhibition of viral replication. It is used in the treatment of HIV infection and chronic hepatitis B infection.

**Figure 1:**
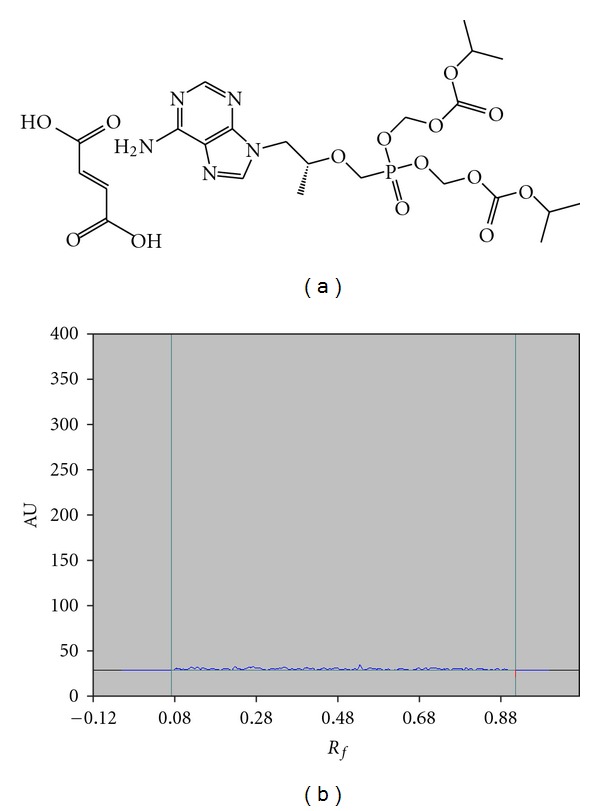
(a) Chemical structure of tenofovir disoproxil fumarate. (b) Representative chromatogram obtained for the placebo.

Chemically tenofovir disoproxil Fumarate (TDF) is fumaric acid salt of the bisisopropoxycarbonyloxymethyl ester derivative of tenofovir. Chemically, it is 9-[(R)-2-[[bis[[(isopropoxycarbonyl)oxy]methoxy]phosphinyl] methoxy]propyl]adenine fumarate (Figure 1(a)). It is not official in any of the pharmacopoeias. This is listed in the Merck Index and Martindale: The complete drug reference [7, 8]. Literature review reveals that several methods have been reported for the estimation of TDF in tablets [9–11], high-performance liquid chromatographic methods [12–16], liquid chromatography-mass spectrometry [17–21], and high-performance thin-layer liquid chromatographic methods (HPTLCs) [22].

An ideal stability-indicating method shall quantify the drug per se and also resolve its degradation products. Thin-layer chromatography has become a part of routine analytical techniques in many product development and analytical laboratories due to its advantages [23–25]. So far, to our present knowledge, no stability-indicating high-performance thin-layer chromatography assay method for the determination of TDF is available in the literature. It was felt necessary to develop a stability-indicating HPTLC method for the determination of TDF as bulk drug and pharmaceutical dosage form and separate the drugs from the degradation products under the ICH-suggested conditions [26]. 

## 2. Experimental

### 2.1. Chemical and Reagents

Pharmaceutical grade TDF (batch no. 481372) working standard was obtained as generous gifts from Ranbaxy Pvt. Ltd., Indore, India. Commercially available Tentide tablets (300 mg) [T-I] were purchased from Ranbaxy Pvt. Ltd., India and Tavin (300 mg) [T-II] from Emcure pharmaceuticals. All chemicals and reagents were of analytical-grade and were purchased from Merck Chemicals, Mumbai, India. 

### 2.2. Instrumentation

#### 2.2.1. For Stress Study

High-precision heating mantel (Remi, India) capable of controlling the temperature within ±1°C was used for generating hydrolytic degradation products. The thermal degradation study was performed using a high-precision hot-air oven (Kumar Scientific Works, Pune, India) capable of controlling the temperature with in ±2°C. Photo-degradation was carried out in a photostability chamber (Thermolab, Scientific Equipment Pvt Ltd.) equipped with lighting system to comply with ICH guideline for photostability condition with white flourescent light exposure for 1.2 million lux hours and integrated near-ultraviolet energy exposure of 200 watts hours/sq·mts (option 2 of the ICH guideline Q1B). At any given time, UV energy and visible illumination were tested using a calibrated lux meter (Lutron, LX-101A).

#### 2.2.2. Chromatography

The HPTLC system consisted of a Camag Linomat 5 semiautomatic spotting device (Camag, Muttenz, Switzerland), a Camag twin-trough chamber (10 cm × 10 cm), Camag winCATS software 1.4.4.6337, and a 100 *μ*L Hamilton syringe. Sample application was done on precoated silica gel 60 F_254_ TLC plates (10 cm × 10 cm). TLC plates were prewashed with methanol and activated at 80°C for 5 min prior to the sample application. Densitometric analysis was carried out utilizing Camag TLC scanner 3.

### 2.3. Preparation of Standard Stock Solutions

A standard stock solution of concentration 1 mg/mL of TDF was prepared in actonitrile. Working standard solutions were prepared by serial dilution of the stock solution with the mobile phase.

### 2.4. General Assay Procedure

#### 2.4.1. Preparation of the Calibration Graph

Working standard solutions containing 300–1500 ng/spot of TDF were prepared by serial dilution of aliquots of the stock solution. Each concentration was applied six times on the TLC plate and the peak areas of TDF were plotted against the corresponding concentration (ng/spot) to obtain the calibration graph.

#### 2.4.2. Analysis of Bulk Substance

The method mentioned above was applied for the determination of the purity of TDF raw material. The percentage recoveries were calculated by referring to the calibration graph previously prepared or applying the regression equation.

#### 2.4.3. Procedure for Stress Testing

A stock solution containing 100 mg TDF in 100 mL acetonitrile was prepared. This solution was used for forced degradation to provide an indication of the stability-indicating ability and specificity of the proposed method. In all degradation studies, the average peak areas of TDF (150 ng/spot) were obtained after application of 6 replicates.

**(a) Hydrolysis**

5 mL of a standard stock solution (1 mg/mL) was mixed with 5 mL 0.01 M HCl at room temperature. The alkaline hydrolysis was carried out by mixing a standard stock solution 5 mL (1 mg/mL) with 0.01 M NaOH (5 mL) kept at room temperature. The solution was then neutralized with 0.01 M NaOH and 0.01 M HCl for the acidic and alkaline degradation, respectively.

**(b) Oxidation**

For the purpose of oxidation studies a mixture of 5 mL standard stock solution and 5 mL hydrogen peroxide (0.3%, v/v) was kept at room temperature and then heated in a boiling water bath for 10 min to completely remove the excess hydrogen peroxide.

**(c) Dry Heat Degradation**

10 mg standard drug as powder was placed in an oven at 50°C for 2 months to study dry-heat degradation.

**(d) Photochemical Degradation**

Photodegradation studies were carried out according to Option 2 of Q1B in ICH guidelines. The stock solution (1 mg/mL) as well as solid drug was exposed to light for an overall illumination of 1.2 million lux/h and an integrated near ultraviolet energy of 200 W hm^−2^ for 8 hrs.

**(e) Neutral Hydrolysis**

To study the degradation behavior of drug in neutral conditions, 5 mL of a standard stock solution (1 mg/mL) was mixed with 5 mL double-distilled water and heated at 80°C for 5 days, and subsequently for 10 days.

#### 2.4.4. Analysis of Dosage Forms

For the analysis of tablets, 20 tablets of each batch T-I and T-II were weighed and finely ground in a mortar. For T-I and T-II, the portion equivalent to 300 mg of TDF was transferred in a 50 mL volumetric flask, 35 mL of acetonitrile was then added, and sonication was done for 45 min with swirling. After sonication, the volume was made up to the mark with the acetonitrile and mixed well. The solution was filtered through Whatman filter paper 41. 

For both T-I and T-II, six determinations were performed.

### 2.5. Optimization of the Stability-Indicating HPTLC Method

HPTLC method was optimized to establish a stability-indicating assay. Both pure and degraded drug solutions were applied to HPTLC plates chromatographed with different mobile phases.

### 2.6. Analytical Method Validation

The developed HPTLC method was validated for linearity, precision, accuracy, sensitivity, robustness, and system suitability. 

#### 2.6.1. Linearity and Range

Accurate quantities from standard solutions were applied on the TLC plate to furnish bands containing 300–1500 ng/spot of TDF. Each amount was applied six times to the plate. The plate was developed in optimized mobile phase and scanned. As it was reported, the correlation coefficient alone is not suitable to prove linearity [27]. So residual plot was generated. 

#### 2.6.2. Precision

The precision of the method was verified by repeatability and intermediate precision studies at a concentration level of 300, 900, and 1500 ng/spot. Repeatability studies were performed by six times analysis on the same day. The intermediate precision of the method was checked by repeating studies on three different days.

#### 2.6.3. Sensitivity

Sensitivity was determined by establishing the limit of detection (LOD) and limit of quantitation (LOQ). LOD and LOQ were calculated as 3.3 and 10 *σ*/S, respectively, where *σ* was the standard deviation of the response (y-intercept) and S was the slope of the calibration curve obtained by injecting a series of dilute solutions with known concentration.

#### 2.6.4. Robustness and System Suitability

Following the introduction of small changes in the optimized mobile phase composition (±0.1 mL for each component), mobile phases having different compositions, for example, chloroform : methanol (8.9 : 1.0 v/v), (9.1 : 1.0 v/v), (9.0 : 0.9 v/v), (9.0 : 1.1 v/v) were tried and densitogram was run. The amount of mobile phase was varied over the range of ±0.1%. The plates were prewashed by methanol and activated at 110°C for 4, 5, 6 min, respectively, prior to chromatography. Time from spotting to chromatography and from chromatography to scanning was varied from +10 min. Robustness of the method was done at a concentration level of 400 ng per band.

#### 2.6.5. Specificity

Tablet matrix without drug components and tablet matrix spiked with drug components were prepared in acetonitrile. The solution of tablet matrix without drug components was made with high-excipient concentration to enable detection of any excipient spots with similar *R*_*f*_ values as the drug components. Spiking of tablets matrix was performed to make a solution with 300, 900, and 1500 ng/spot.

#### 2.6.6. Accuracy

Accuracy of the method was tested by applying the method to drug sample to which known amounts of TDF standard powder corresponding to 80, 100, and 120% of label claim had been added (standard addition method), mixed, and the powder was extracted and analyzed by running chromatograms in optimized mobile phase. These mixtures were analyzed by the proposed method. The experiment was performed in triplicate and recovery (%) was calculated.

#### 2.6.7. Analysis of Marketed Formulation

The contents of drug in tablets were determined by the proposed method using the calibration curve.

#### 2.6.8. Solution Stability

The solution stability of TDF was carried out by leaving the test solution in tightly capped volumetric flasks at room temperature for 24 h and assayed at 6 h interval against the freshly prepared standard solution. The %RSD of assay of TDF was calculated for the study period during mobile phase and solution stability experiments. 

## 3. Results and Discussion

### 3.1. Method Development and Optimization

**Figure 2:**
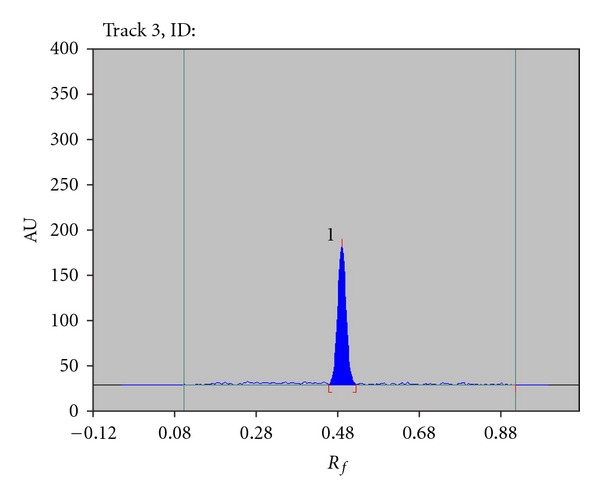
Densitogram of standard tenofovir disoproxil fumarate 100 ng/spot; Peak 1 (*R*_*f*_: 0.49 ± 0.03), mobile phase-chloroform : methanol (9.0 : 1.0 v/v).

Different proportions of acetone, toluene, methanol, and chloroform were tried for selection of mobile phase. Ultimately, chloroform : methanol (9.0 : 1.0 v/v) was found to be optimum (Figure 2) at *R*_*f*_  0.49 ± 0.03. In order to reduce the necklace effect, TLC chamber was saturated for 30 min using saturation pads. The mobile phase was run up to a distance of 8 cm; which takes approximately 20 min for complete development of the TLC plate and scanning wavelength was 260.

### 3.2. Detection of Degradation Products by HPTLC

#### 3.2.1. Hydrolysis

Acid and alkaline degradation of TDF was performed in 1 : 1 acetonitrile—0.01 M HCl and NaOH. TDF was highly susceptible to attack by HCl and NaOH. Complete degradation occurred immediately after addition of HCl and NaOH at room temperature.

#### 3.2.2. Oxidation

The drug was found to be unstable to oxidative degradation. In 1 : 1 acetonitrile, 0.3% H_2_O_2_ complete degradation occurs immediately at room temperature.

#### 3.2.3. Dry Heat Degradation Product

There was no significant degradation of solid TDF on exposure to dry heat at 50°C for 2 months, which indicated that drug was stable against thermal stress.

#### 3.2.4. Photolysis

**Figure 3:**
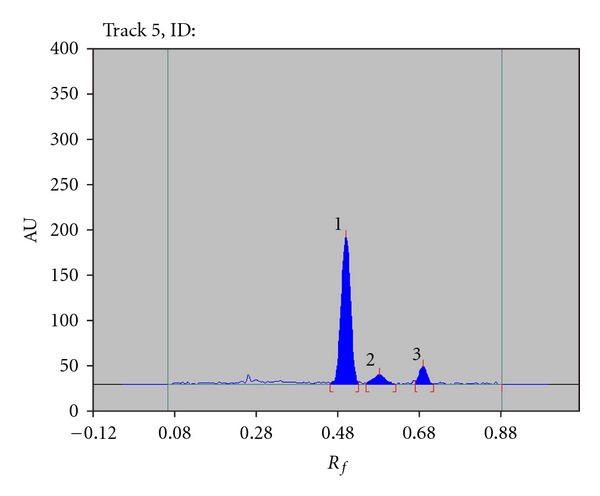
Densitogram of the photodegradation product 150 ng/spot; condition: white fluorescent and integrated near-ultraviolet energy at 1.2 million lux hours and 200 watts hours/sq·mts for 8 h; Peak 1 (tenofovir disoproxil fumarate, *R*_*f*_: 0.50), Peak 2 (degraded, *R*_*f*_: 0.58), and Peak 3 (degraded, *R*_*f*_: 0.72).

TDF was degraded in photochemical degradation after exposing drug to a combination of white fluorescent and integrated near-ultraviolet energy at 1.2 million lux hours and 200 watts hours/sq·mts, respectively, for 8 h forming two major degradation products at 0.58 and 0.72 (Figure 3).

#### 3.2.5. Neutral Degradation

TDF under neutral hydrolysis did not give rise to the presence of degradants as the peak area remained constant which indicated drug stability under the conditions investigated.

### 3.3. Validation

#### 3.3.1. Linearity

**Figure 4:**
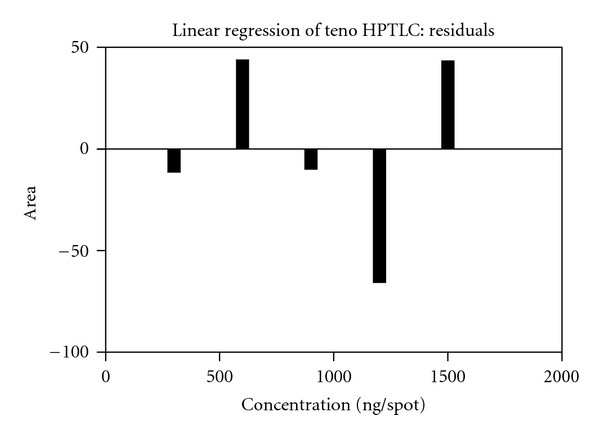
Residual plot of tenofovir disoproxil fumarate.

Good linearity was observed in the concentration range of 300–1500 ng/spot of TDF. The data was subjected to statistical analysis using a linear regression model; the result shows that, within the concentration range mentioned above, there was an excellent correlation between peak areas, and concentrations of drug intercept and slope were found to be 13.37 and 5.70, respectively, with correlation coefficient of 0.999. The relationship between the concentration of each of TDF and peak area of the spot was investigated. The linear relationship was tested and found to be linear, indicating good correlation (Figure 4).

#### 3.3.2. Precision

Precision was evaluated by carrying out six independent sample preparations of a single lot of formulation. Percentage relative standard deviation (%RSD) was found to be less than 2% for repeatability and intermediate variations as shown in Table 1.

#### 3.3.3. LOD and LOQ

The LOD and LOQ values were found to be 8 and 25 ng/spot.

#### 3.3.4. Specificity

The densitogram of the solution of the nonspiked tablet matrix did not show any spots. On the other hand, the densitogram of the solution of tablet matrix spiked with TDF showed clear, compact spot. Moreover, no other spots eluted besides the active compounds. Therefore, the method was considered specific (Figure 1(b)).

#### 3.3.5. Robustness of the Method

The robustness of the method was determined by variations in mobile phase composition (±0.1 mL for each component), amount of mobile phase (±1%), time from spotting to chromatography, and from chromatography to scanning (+10 min). One factor at a time was changed at a concentration level of 400 ng/spot, to study the effect on the peak area of the drugs. The method was found to be unaffected by small changes with %RSD for all the parameters less than 2%, indicating that the method is robust shown in Table 2.

#### 3.3.6. Solution Stability Study

No additional peak was found in the densitogram of sample from solution stability. The results from solution-stability and mobile-phase-stability experiments confirmed that standard solutions and solutions in the mobile phase were stable up to 24 h for assay and related substances analysis as shown in Table 3.

#### 3.3.7. Recovery Studies

Good recoveries of the TDF were obtained at various added concentrations for T-I and T-II as shown in Table 4.

#### 3.3.8. Analysis of a Commercial Formulation

Experimental results of the amount of TDF in tablets, expressed as a percentage of label claims, were in good agreement with the label claims thereby suggesting that there is no interference from any of the excipients which are normally present in tablets. Two different brands of fixed dose combination tablets were analyzed using the proposed procedures as shown in Table 5. 

## 4. Conclusion

This study showed that tenofovir disoproxil fumarate was found to be unstable under acidic, alkaline, and oxidative conditions as it degradaed completely, but it was found that it is labile to photolysis and that complete separation of degradants was carried out using stability-indicating HPTLC method. Tenofovir disoproxil fumarate was observed to be stable when exposed to neutral condition and dry heat. The developed HPTLC method proved to be simple, accurate, precise, and specific. Hence, it is recommended for industrial analysis of drug and degradation products obtained from stability procedures.

##  Conflict of Interests

The authors declared that they do not have anything to disclose regarding funding or conflict of interests with respect to this paper.

## Figures and Tables

**Table 1:** Precision of HPTLC method^a^.

Compound	Repeatability		Intermediate	
	Mean% assay	%RSD	Mean% assay	%RSD
TDF	100.30	1.05	100.61	1.26

^a^ *n* = 6, (300, 900, 1500 ng/spot).

**Table 2:** Robustness testing^a^.

Parameters	SD of peak area	%RSD
Mobile phase composition (±0.1 mL)	29.19	1.12
Amount of mobile phase (±5%)	15.22	1.01
Plate pretreatment (4, 5, 6 min)	9.51	0.07
Time from spotting to chromatography (+10 min)	2.03	0.03
Time from chromatography to scanning (+10 min)	0.73	0.05

^a^ *n* = 6.

**Table 3:** Stability of drug in sample solution^a^.

Time of analysis (h)	SD	%RSD
6	9.37	0.22
12	9.02	0.38
18	8.93	0.01
24	8.62	0.72

^a^(*n* = 6).

**Table 4:** Recovery studies^a^.

Label claim	Amount of drug added (%)	Total amount of drug present *μ*g/mL	Amount found *μ*g/mL	% Recovery ± SD
		T-I		

300 mg	80	10800	10733.04	99.38 ± 0.82
	100	12000	11941.2	99.51 ± 0.23
	120	13200	13101	99.25 ± 0.63

		T-II		

300 mg	80	10800	10725.48	99.31 ± 0.22
	100	12000	11980.8	99.84 ± 0.30
	120	13200	13082.52	99.11 ± 0.75

^a^ *n* = 6.

**Table 5:** Applicability of the HPTLC method for the analysis of the pharmaceutical formulations.

Label claim (mg)	Sample	Drug Content (%)	%RSD
300	T-I	99.13	1.03
	T-II	99.37	0.84

## References

[1] International Conference on Harmonization Stability testing of new drug substances and products Q1A (R2).

[2] Singh S, Bakshi M (2000). Guidance on conduct of stress tests to determine inherent stability of drugs. *Pharmaceutical Technology Online*.

[3] ICH Guidelines on photostability testing of newdrug substances and products.

[4] Singh S, Bakshi M (2002). Development of validated stability-indicating assay methods—critical review. *Journal of Pharmaceutical and Biomedical Analysis*.

[5] Singh S, Singh B, Bahuguna R, Wadhwa L, Saxena R (2006). Stress degradation studies on ezetimibe and development of a validated stability-indicating HPLC assay. *Journal of Pharmaceutical and Biomedical Analysis*.

[6] Carstensen JT, Rhodes CT (2000). *Drug Stability Principles and Practices*.

[7] Sweetman SC (2005). *Martindale-The Complete Drug Reference*.

[8] Budawari S (2001). *The Merck Index*.

[9] Patel S, Baghel US, Rajesh P, Prabhakar D, Engla G, Nagar PN (2009). Spectrophotometric method development and validation for simultaneous estimation of Tenofovir disoproxil fumarate and Emtricitabine in bulk drug and tablet dosage form. *International Journal of Pharmaceutical and Clinical Research*.

[10] Pendela M, Getu WK, Van den Mooter G, Baert L, Hoogmartens J, Adams E (2011). LC assay for a HIV tablet containing emtricitabine, tenofovir disoproxil fumarate and rilpivirine. *Chromatographia*.

[11] Raju NA, Begum S (2008). Simultaneous RP-HPLC method for the estimation of the Emtricitabine,Tenofovir disoproxil fumarate and Efavirenz in tablet dosage forms. *Research Journal of Pharmacy and Technology*.

[12] Seshachalam U, Rajababu B, Haribabu B, Chandrasekhar KB (2008). Enantiomeric separation of tenofovir on an achiral C_18_ column by HPLC using L-phenylalanine as a chiral mobile phase additive. *Journal of Liquid Chromatography and Related Technologies*.

[13] Jullien V, Tréluyer JM, Pons G, Rey E (2003). Determination of tenofovir in human plasma by high-performance liquid chromatography with spectrofluorimetric detection. *Journal of Chromatography B*.

[14] Rezk NL, Crutchley RD, Kashuba ADM (2005). Simultaneous quantification of emtricitabine and tenofovir in human plasma using high-performance liquid chromatography after solid phase extraction. *Journal of Chromatography B*.

[15] Sentenac S, Fernandez C, Thuillier A, Lechat P, Aymard G (2003). Sensitive determination of tenofovir in human plasma samples using reversed-phase liquid chromatography. *Journal of Chromatography B*.

[16] Kandagal PB, Manjunatha DH, Seetharamappa J, Kalanur SS (2008). RP-HPLC method for the determination of tenofovir in pharmaceutical formulations and spiked human plasma. *Analytical Letters*.

[17] Barkil ME, Gagnieu MC, Guitton J (2007). Relevance of a combined UV and single mass spectrometry detection for the determination of tenofovir in human plasma by HPLC in therapeutic drug monitoring. *Journal of Chromatography B*.

[18] Bezy V, Morin P, Couerbe P, Leleu G, Agrofoglio L (2005). Simultaneous analysis of several antiretroviral nucleosides in rat-plasma by high-performance liquid chromatography with UV using acetic acid/hydroxylamine buffer: test of this new volatile medium-pH for HPLC-ESI-MS/MS. *Journal of Chromatography B*.

[19] Delahunty T, Bushman L, Robbins B, Fletcher CV (2009). The simultaneous assay of tenofovir and emtricitabine in plasma using LC/MS/MS and isotopically labeled internal standards. *Journal of Chromatography B*.

[20] Delahunty T, Bushman L, Fletcher CV (2006). Sensitive assay for determining plasma tenofovir concentrations by LC/MS/MS. *Journal of Chromatography B*.

[21] King T, Bushman L, Kiser J (2006). Liquid chromatography-tandem mass spectrometric determination of tenofovir-diphosphate in human peripheral blood mononuclear cells. *Journal of Chromatography B*.

[22] Joshi M, Nikalje AP, Shahed M, Dehghan M (2009). HPTLC method for the simultaneous estimation of emtricitabine and tenofovir in tablet dosage form. *Indian Journal of Pharmaceutical Sciences*.

[23] Kulkarni SP, Amin PD (2000). Stability indicating HPTLC determination of timolol maleate as bulk drug and in pharmaceutical preparations. *Journal of Pharmaceutical and Biomedical Analysis*.

[24] Thoppil SO, Cardoza RM, Amin PD (2001). Stability indicating HPTLC determination of trimetazidine as bulk drug and in pharmaceutical formulations. *Journal of Pharmaceutical and Biomedical Analysis*.

[25] Makhija SN, Vavia PR (2001). Stability indicating HPTLC method for the simultaneous determination of pseudoephedrine and cetirizine in pharmaceutical formulations. *Journal of Pharmaceutical and Biomedical Analysis*.

[27] Ferenczi-Fodor K, Végh Z, Nagy-Turák A, Renger B, Zeller M (2001). Validation and quality assurance of planar chromatographic procedures in pharmaceutical analysis. *Journal of AOAC International*.

